# Supplementary figures and images for: Generation of the short TRIM32 isoform is regulated by Lys 247 acetylation and a PEST sequence
Source: PLoS One. 2021 May 17;16(5):e0251279. doi: 10.1371/journal.pone.0251279 (PMC8128265; doi:10.1371/journal.pone.0251279)

## Supporting Information S1 Fig 1

**A**

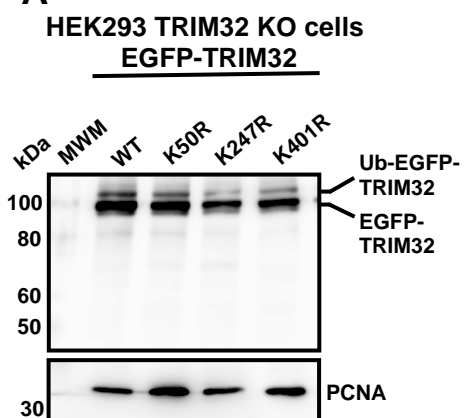

**B**

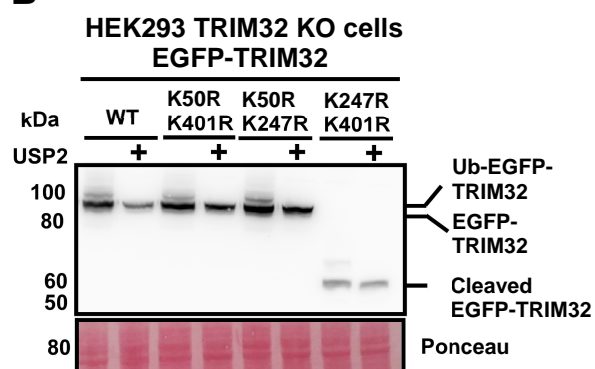

Supplement: S1 Fig — A. Western Blot analysis of HEK293 FlpIn TRIM32 KO cells transiently transfected with expression plasmids for EGFP-TRIM32WT, EGFP-TRIM32K50R, EGFP-TRIM32K247R, or TRIM32K401R. The bands representing EGFP-TRIM32 and auto-ubiquitylated EGFP-TRIM32 are indicated to the right. PCNA represents the loading control. B. Western Blot analysis of cell extracts from HEK293 FlpIn TRIM32 KO cells transiently transfected with expression plasmids for EGFP-TRIM32WT, EGFP-TRIM32K50R/K401R, EGFP-TRIM32K50R/K247R or EGFP-TRIM32K247R/K401R. Expression plasmid for mCherry-USP2 is co-transfected where indicated. The bands representing EGFP-TRIM32, auto-ubiquitylated EGFP-TRIM32 and cleaved EGFP-TRIM32 are indicated to the right Ponceau represents the loading control. (PDF) [file pone.0251279.s001.pdf]
