# Supplementary material for: Generation of the short TRIM32 isoform is regulated by Lys 247 acetylation and a PEST sequence
Source: PLoS One. 2021 May 17;16(5):e0251279. doi: 10.1371/journal.pone.0251279 (PMC8128265; doi:10.1371/journal.pone.0251279)
Supplement: S1 Raw images — (PDF) [file pone.0251279.s002.pdf]

Raw images of the blots presented in Figs 1, 2 and 4

The order of the lanes is the same as in the Figs, and the size of the MW marker bands are as displayed in the Figs. The blots were developed as describedin the material and methods section.

The Figs corresponding to the raw data file is indicated below the image.

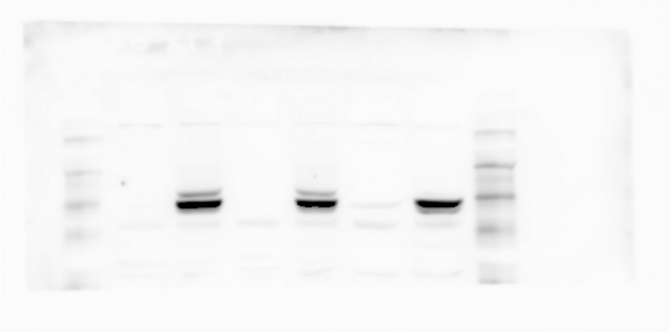

Fig. 1A, anti-GFP antibody

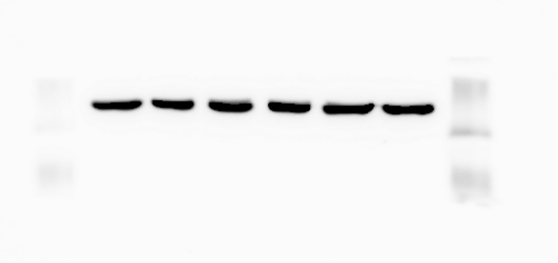

Fig. 1A, anti-actin antibody

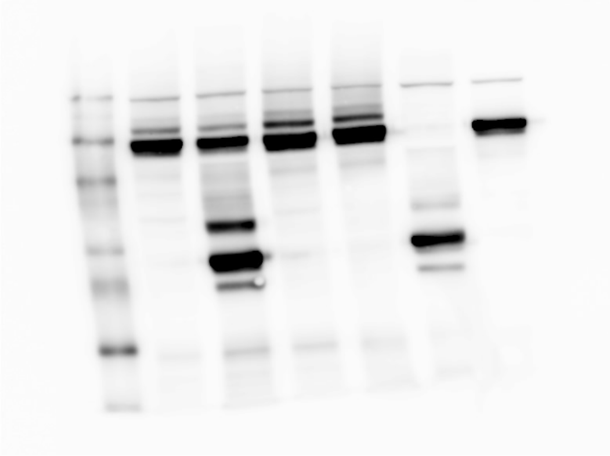

Fig. 1D, anti-GFP antibody

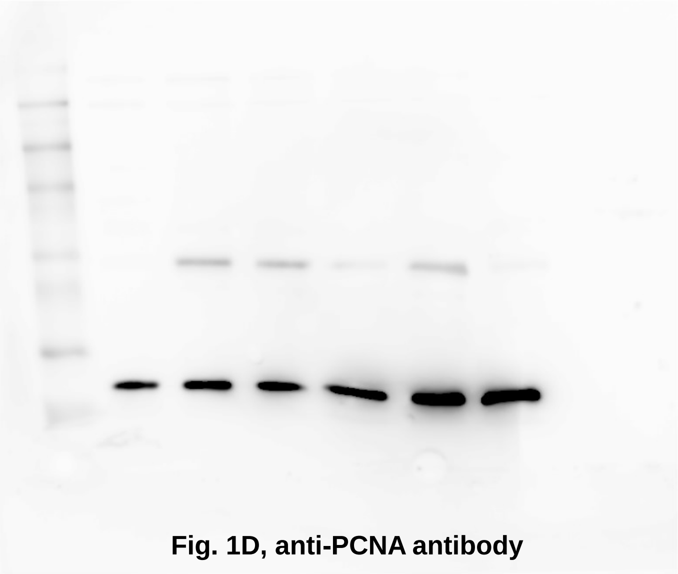

Fig. 1D, anti-PCNA antibody

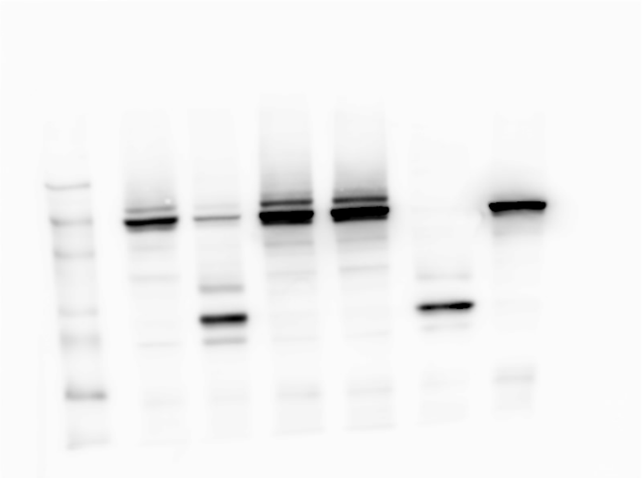

Fig. 1E, anti-TRIM32 antibody

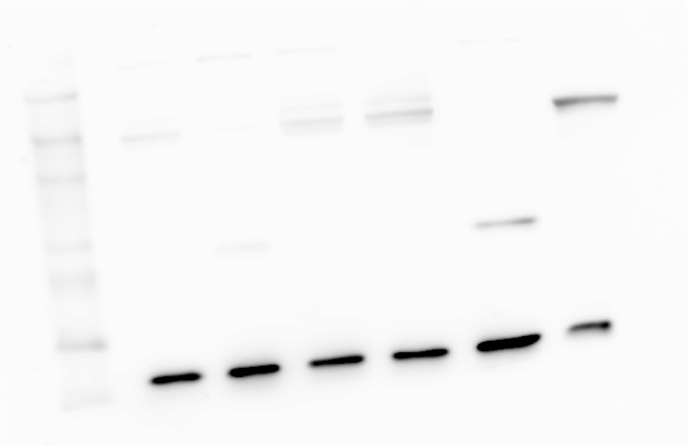

Fig. 1E, anti-PCNA antibody

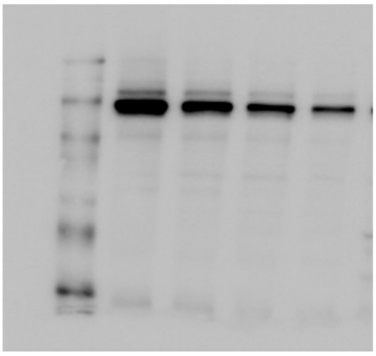

Fig. S1A, anti-GFP antibody

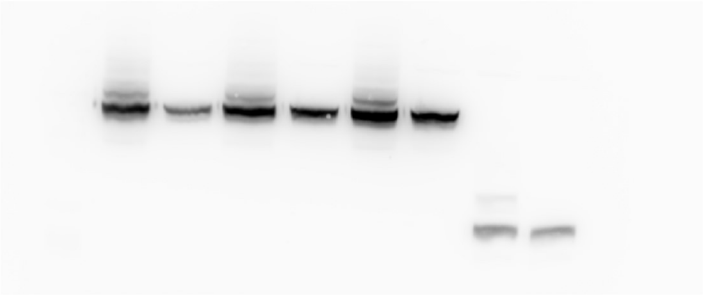

Fig. S1B, anti-GFP antibody

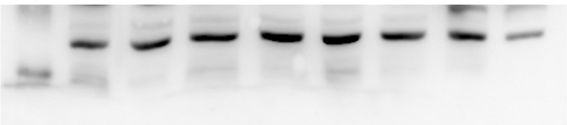

Fig. S1B, anti-Actin antibody

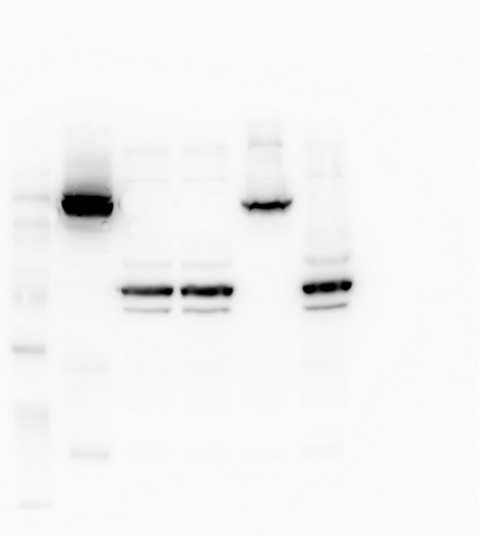

Fig. 2B, anti-Trim32 antibody

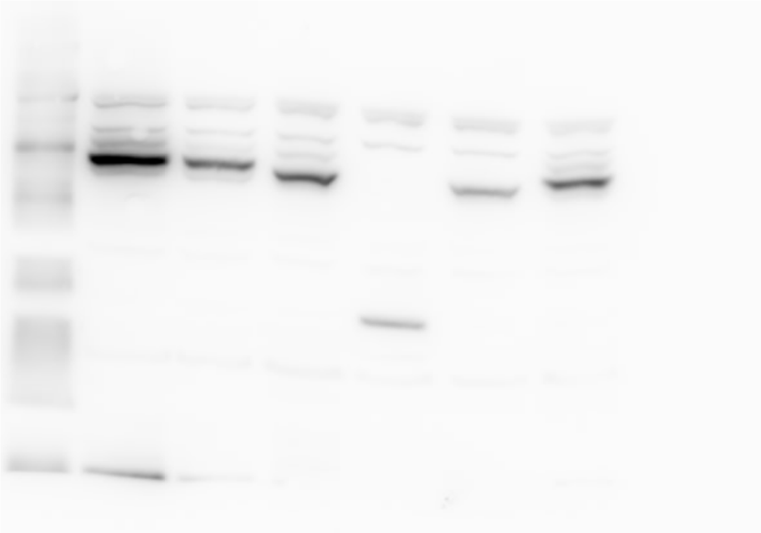

Fig. 4B, anti-Trim32 antibody

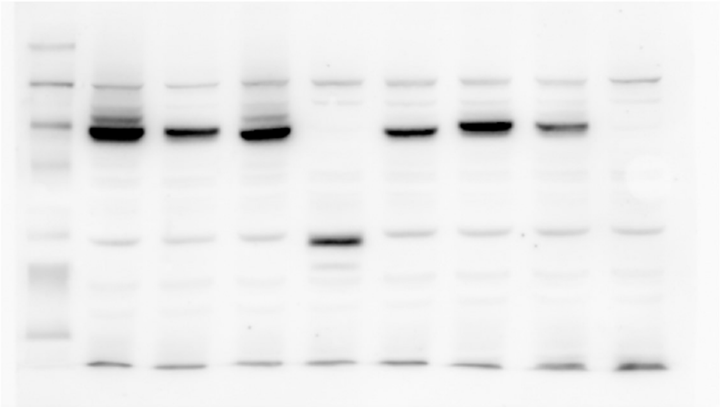

Fig. 4C, anti-TRIM32 antibody

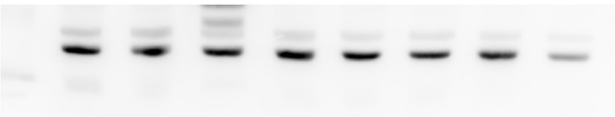

Fig. 4C, anti-Actin antibody

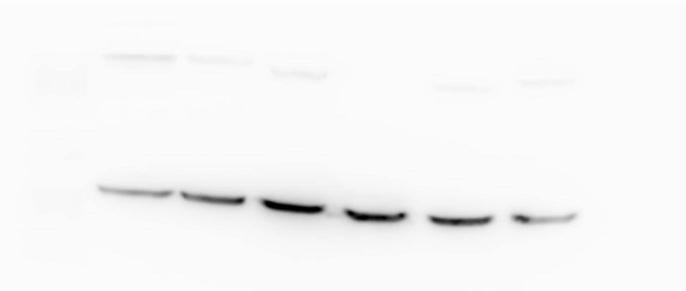

Fig. 4B, anti-tubulin antibody
